# INTRODUCING ARTIFICIAL COMPANIONS TO USERS WITH DEMENTIA IN UNREGULATED MARKETS: OPPORTUNITIES VS. ETHICAL ISSUES

**DOI:** 10.1093/geroni/igz038.1386

**Published:** 2019-11-08

**Authors:** Elena Portacolone, Jodi halpern, Jay Luxenberg, Krista Harrison, Kenneth Covinsky

**Affiliations:** 1 University of California San Francisco, San Francisco, California, United States; 2 University of California Berkeley, Berkeley, California, United States; 3 On Lok, San Francisco, California, United States; 4 UCSF, San Francisco, California, United States

## Abstract

Because of the high costs of providing long-term care, artificial companions are increasingly considered an opportunity to provide support to older adults with cognitive impairment while saving costs. Artificial companion can comfort and inform, thus inducing a sense of being in a relationship. Sensors and algorithms usually allow these applications to exude a life-like feel. The explosion of these technologies has created a “cultural lag” between their rapid commercial introduction and the slower evolution of regulations. An outcome of this cultural lag is a tension between the potential of artificial companions to support users and a series of unresolved ethical issues related to the fact that users might lack the capacity to fully understand the implications of using these technologies. Specific challenges of deception, surveillance, consent and social isolation are raised by the introduction of these technologies in users with cognitive impairment. The case study of a sophisticated artificial companion commercially available in the United States lends the opportunity to examine the tension between the potential of this technologies vs. unresolved ethical issues. This companion is an avatar on an electronic tablet that is displayed as a dog or a cat. Whereas artificial intelligence guides most artificial companions, this application is a hybrid of robots and human beings because it also relies on technicians “behind” the on-screen avatar, who via surveillance, interact with users. We conclude with a call to develop regulations promoting artificial companions as “human-driven technologies,” i.e. technologies focused on truly empowering users according to their cognitive abilities.

